# The roles of MASPIN expression and subcellular localization in non-small cell lung cancer

**DOI:** 10.1042/BSR20200743

**Published:** 2020-05-26

**Authors:** Xiao-Fei Wang, Bo Liang, Da-Xiong Zeng, Wei Lei, Cheng Chen, Yan-Bin Chen, Jian-An Huang, Ning Gu, Ye-Han Zhu

**Affiliations:** 1 Department of Pulmonary and Critical Care Medicine, The First Affiliated Hospital of Soochow University, Suzhou, China; 2 Nanjing University of Chinese Medicine, Nanjing, China; 3 Nanjing Hospital of Chinese Medicine Affiliated to Nanjing University of Chinese Medicine, Nanjing, China

**Keywords:** MASPIN, non-small cell lung cancer, prognosis, SERPINB5

## Abstract

Accumulating studies have confirmed that mammary serine protease inhibitor (MASPIN) plays an essential role in non-small cell lung cancer (NSCLC). However, results are still controversial or inconsistent. In the present study, we attempted to identify the clinical significance of MASPIN and its potential molecular roles in NSCLC. The correlation of MASPIN with prognosis and clinicopathological characteristics was assessed by meta-analysis. Additionally, the potential molecular mechanisms of MASPIN in NSCLC was also investigated through several online databases. A total of 2220 NSCLC patients from 12 high quality studies were included and the results indicated that up-regulated MASPIN nucleus and cytoplasm expression was associated with poor overall survival (OS) (hazard ratio (HR) = 1.43, 95% confidence interval (CI) = 1.01–2.04, *P*<0.05), elevated MASPIN cytoplasm expression was associated with poor OS (HR = 1.45, 95% CI = 1.01–2.07, *P*<0.05), disease-free survival (DFS) (HR = 1.95, 95% CI = 1.31–2.88, *P*=0.001), and disease-specific survival (DSS) (HR = 2.17, 95% CI = 1.18–3.99, *P*=0.013). MASPIN both nucleus and cytoplasm location were associated with clinicopathological characteristics. Bioinformatics analysis validated the above results and suggested that human serpin family B member 5 (*SERPINB5*) hypomethylated levels were negatively correlated with its mRNA expression. Bioinformatics analysis also revealed the 85 most frequently altered neighboring genes of *SERPINB5*, and gene ontology (GO) and Kyoto Encyclopedia of Genes and Genomes (KEGG) pathway enrichment analysis revealed 20 GO terms and 3 KEGG pathways with statistical significance. MASPIN had a statistically negative correlation with NSCLC prognosis, functioning as an oncoprotein by hypomethylation and influencing specific pathways involving the 85 genes identified herein. MASPIN might be a promising prognostic signature in NSCLC.

## Introduction

The mammary serine protease inhibitor (MASPIN), encoded by the human serpin family B member 5 (*SERPINB5*) gene, was first reported in 1994 as a tumor suppressor [1]. MASPIN is highly expressed in normal mammary epithelial cells but is often down-regulated or not expressed in the progression of breast malignant tumors [1,2]. Advanced research found that MASPIN inhibits cell invasion, promotes apoptosis, and inhibits angiogenesis, so it is considered to have class II tumor-suppressive properties [3]. MASPIN localizes in not only primarily cytoplasm (in the mammary epithelial cells), but also nucleus (in the myoepithelial cells), secretory vesicles, and cell surface [4]. MASPIN subcellular localization appears linked to its biological functions [5], and its nuclear localization indicates a more favorable prognosis in human malignancies, such as breast cancer [6], pancreatic cancer [4], and ovarian carcinoma [7]. This phenomenon also appears in non-small cell lung cancer (NSCLC) [8], which is one of the most familiar malignant tumors [9,10] and can be mainly divided into lung squamous cell carcinoma (LUSC) and lung adenocarcinoma (LUAD), and lung large cell carcinoma, pathologically [11]. One study shows emaciated cytoplasmic MASPIN expression could consider as an independent positive predictor in primary NSCLC [12], whereas another study shows that dominant nuclear expression was related to positive prognosis in resectable NSCLC [13]. This paradoxical localization and expression of MASPIN might have cell-specific characteristics [14] and play different roles in NSCLC. Moreover, MASPIN may have an ability to limit malignancy cell phenotypic plasticity and affect the cancer cell response to drugs [15], so it might be a valuable marker and potential therapeutic agent [5]. Thus, we conducted present study to clarify the roles of MASPIN expression and subcellular localization in NSCLC.

## Materials and methods

### Studies search and selection

We performed a systematic search on Medline, Embase, and Web of Science (WOS) for relevant published studies from inception to 31 January 2019, with language restricted to English. The following keywords were used in different combinations: ‘Carcinoma’, ‘Non-Small-Cell Lung’, ‘MASPIN’, ‘SERPIN-B5’, ‘Prognosis’. Search strategies in Medline were available in Supplementary Table S1.

Inclusion criteria for studies: (1) participants were pathologically diagnosed with NSCLC; (2) the expression and subcellular localization were detected by immunohistochemistry; (3) patients were divided into high and low expression groups according to MASPIN expression and the relationship between MASPIN and NSCLC prognosis (such as overall survival (OS), disease-free survival (DFS), progression-free survival (PFS), and disease-specific survival (DSS)) and clinicopathological characteristics were described; (iv) relevant data were sufficient to obtain hazard ratio (HR) and corresponding 95% confidence interval (CI); (v) cohort studies. Exclusion criteria included reviews, case reports, comments, expert opinions, meta-analysis, and conference summaries. When studies or data duplication were found, only the latest or most complete studies were included.

### Data extraction and management

Citations were screened at the title and abstract level by two independent reviewers, and the full text was retrieved for those potentially eligible. For studies that did not provide HRs and 95% CIs directly, we estimated them through indirect methods [16]. If only Kaplan–Meier curves were available, HRs were extracted from graphics by Engauge Digitizer [17]. One reviewer extracted data of interest into a predesigned excel spreadsheet. Then, another two reviewers checked received data and contradictory cases were resolved by the final author.

### Quality assessment

Two reviewers critically and independently assessed included studies using the Newcastle–Ottawa Scale (NOS) [18,19]. The assessments include selection, comparability, and outcome. The studies were divided into three grades: low, medium, and high, with NOS scores ranging from 0 to 3, 4 to 6, and 7 to 9, respectively. All data were finally shown in the predesigned excel spreadsheet and discrepancies, contradictories were resolved by the third author.

### Data synthesis and analysis

The STATA, statistical software for data science, was adopted to synthesize and analyze data. Similar to the method described previously [20], heterogeneity was assessed by *I^2^* tests. If the data were substantially homogeneous, a synthesis was directly conducted with the fixed-effect model. Otherwise, a synthesis was directly conducted with the random-effect model, successively subgroup analysis was performed. Sensitivity analysis was additionally conducted to investigate the robustness of our results. At last, the funnel plot and Egger’s tests were applied to assess for publication bias. Later, the different subcellular location was also analyzed.

### Bioinformatics analysis

The *SERPINB5* mRNA and protein expression between NSCLC and normal tissue were studied using UALCAN [21] and Human Pathology Atlas [22], respectively. The association between *SERPINB5* and OS or PFS and the clinicopathological significances of *SERPINB5* in NSCLC were all conducted in the Kaplan–Meier plotter [23]. The patients were grouped by median *SERPINB5* expression and HR with 95% CI were calculated. In addition, MethHC [24] was utilized to compare the *SERPINB5* promoter region methylation between NSCLC and normal tissue as well as the relationship between mRNA expression and methylation in NSCLC patients. One-way ANOVA was used to analyze differential expression. Moreover, the genes co-expressed with *SERPINB* in TCGA-LUAD were identified using the cBioPortal [25]. Only those genes with Spearman’s correlation coefficients of more than 0.4 were identified as *SERPINB*-related genes. Finally, *SERPINB*-related genes were loaded into DAVID [26,27] for gene ontology (GO) and Kyoto Encyclopedia of Genes and Genomes (KEGG) pathway analysis, and the illustration of the biological significance was performed using the *clusterProfiler* package in R [28].

## Results

### Characteristics of eligible studies

**Figure 1:**
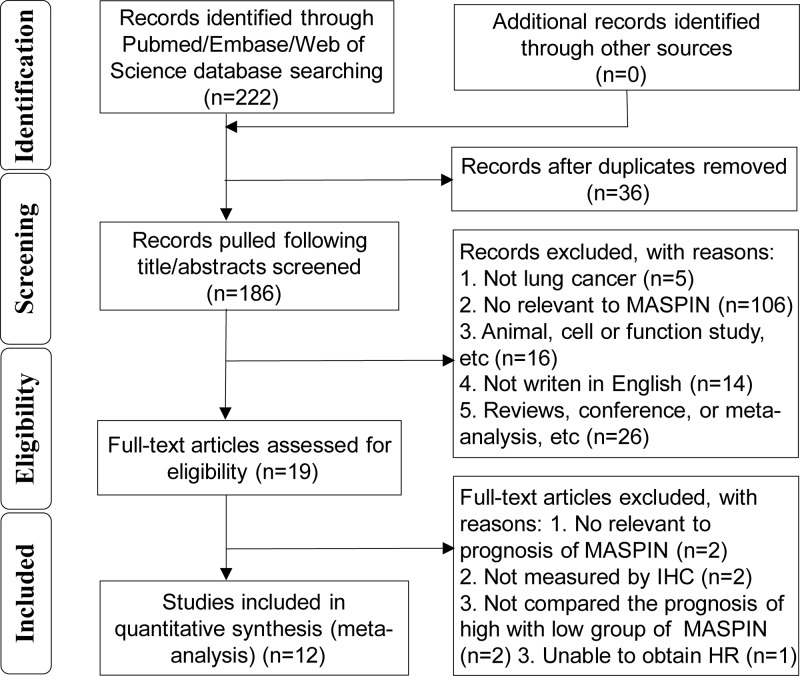
Flow diagram of the selection process in this meta-analysis

The primary search of all sources yielded 222 articles eligible for identification. A total of 186 title/abstracts were screened for eligibility after removing 36 duplicates. Of these, 19 full-text articles were assessed for eligibility. Finally, a total of 12 studies [12,29–39] were included in the present study (Figure 1).

Twelve studies comprising 2220 patients contained information regarding OS, PFS, DFS, and DSS. All included studies were published in 2005 and later, and more than half of the studies were conducted in Japan [12,31–35,39], only three studies involved LUSC or LUAD [31,33,34]. Only one study did not report gender composition [37]. Two studies did not report the median age [31,37], and the median age of the included individuals was approximately 65 years old or so. Only two studies had no staging information for NSCLC [36,37]. As for subcellular localization, three studies [12,30,34] only involved cytoplasm, while others included both cytoplasm and nucleus. Some HRs were extracted directly from corresponding studies [29–35], and some were extracted from corresponding curve [12,32,35–39], and some were not reported [29,32,38]. The included studies also had different definitions of the cut-off value. NOS scores of all eligible studies were greater than 7. The main characteristics of the included studies are shown in Table 1.

**Table 1:** Main characteristics of eligible studies

Author, year	Country	Type	No. (M/F)	Median age	Stage	Location	Cut-off	Positive (%)	Median follow-up	Outcome	HR estimation	NOS
Hirai, 2005	Japan	A94/S38	132 (90/42)	65.5	I-II78/III54	C	≥40%	73 (55.3%)	NR (1–42 m)	Negative (NSCLC+C)	OS curve	8
Nakagawa, 2006	Japan	A116/S76/O18	210 (151/59)	62.6	I-II142/III68	T	intensity+-++	73 (34.8%)	NR (1–60 m)	NO (NSCLC+T)/Positive (S+T)	OS NR/OS R	8
Woenckhaus, 2007	Germany	A74/S209/O69	487 (NR)	NR	NR	T (C+N)	≥10%	352 (72.3%)	27 m (0–200 m)	NO (NSCLC+C)/NO (NSCLC+N)	OS curve/OS curve	7
Zheng, 2008	China+Japan	A86/S37/O32	155 (105/50)	69.5	I-II115/III-IV37	T (C+N)	>5%	91 (58.7%)/21 (13.5%)	20.4 m (1–144 m)	NO (NSCLC+C)/Positive (A+C)	OS curve/OS curve	7
Takanami, 2008	Japan	A105/S70/O6	181 (130/51)	65.5	I-II126/III55	T	≥5%	74 (40.9%)	75.2 m (60–108 m)	NO (NSCLC+T)/Positive (S+T)	PFS, OS curve/PFS, OS R	8
Bircan, 2010	Turkey	A18/S28	46 (42/4)	61.4	II3/III-IV37	C	>5%	39 (84.8%)	261.8 d (10–800 d)	NO (NSCLC+C)	OS R	7
Berardi, 2012	Italy	A192/S172/O75	439 (374/65)	68	I-II333/III106	T (C+N)	>5%	284 (67%)/79 (18.6%)	42.5 m (10.6–87.2 m)	Negative (NSCLC+C)/Positive (NSCLC+N)	OS NR/OS R	7
Wang, 2014	China	A71/S23/O4	98 (60/38)	63	NR	T	intensity++	26 (26.5%)	42.8 m (6.87–69 m)	NO (NSCLC+T)	DFS curve	8
Takagi, 2015	Japan	A110	110 (55/55)	67.9	I-II101/III9	C	>10%	27 (24.5%)	71 m (6–88 m)	Negative (A+C)	DFS, OS R	7
Yaman, 2015	Turkey	A45/S23/O12	80 (71/9)	59	I-II50/III-IV30	T	>5%	26 (32.1%)	41.4 m (1–91 m)	NO (NSCLC+T)/Negative (A+T)	OS NR/OS Curve	8
Matsuoka, 2016	Japan	S101	101 (92/9)	NR	I-II90/III11	T (C+N)	>10%	25 (24.6%)	50 m (2–119 m)	Negative (S+C)	DFS, DSS R	9
Ohno, 2018	Japan	A181	181 (87/94)	69.7	I181	T (C+N)	>10%	45 (24.9%)	74 m (12–151 m)	Negative (A+C)	DFS, DSS R	9

Abbreviations: A, adenocarcinoma; C, cytoplasm; F, female; m, month; M, male; No., number; N, nucleus; NR, not reported; O, other type non-small lung cancer; R, reported in text; S, squamous cell carcinoma; T, total, T = C+N.

### Association between MASPIN and NSCLC prognosis

**Figure 2:**
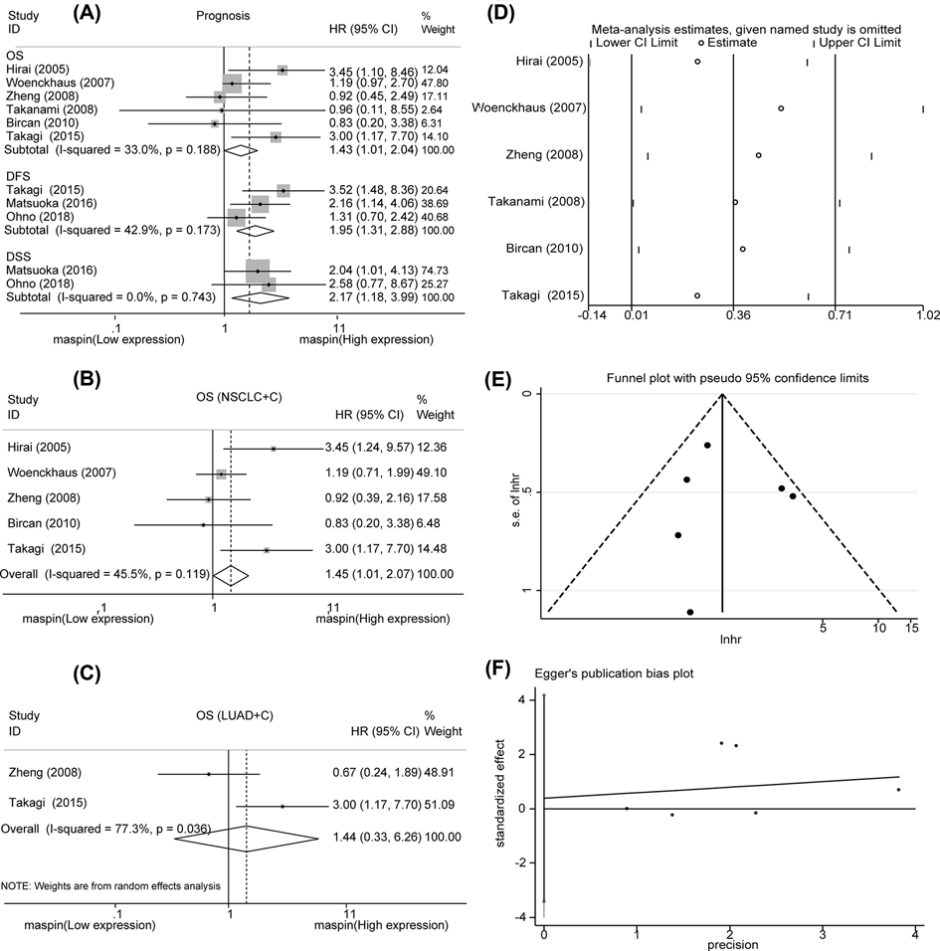
Forest plot of the HR for the correlation between survival rate and MASPIN expression (**A**) The association of MASPIN expression with OS, PFS, DFS, and DSS. (**B,C**) The association of MASPIN expression with OS by subgroup analysis. (**D**) Sensitivity analysis of OS. (**E**) Publication bias analysis of OS by funnel plot. (**F**) Publication bias analysis of OS by Egger’s test. Abbreviations: C, cytoplasm; CI, confidence interval; N, nucleus; T, total, T = C+N.

The differences in MASPIN expression and subcellular localization were analyzed in six studies. As a result, we found that up-regulated MASPIN nucleus and cytoplasm expression was associated with poor OS in NSCLC (HR = 1.43, 95% CI = 1.01–2.04, *P*<0.05) without obvious heterogeneity (*I*^2^ = 33.00%, *P*_h_<0.05, Figure 2A). The results also showed that elevated MASPIN cytoplasm expression was associated with poor DFS in NSCLC (HR = 1.95, 95% CI = 1.31–2.88, *P*=0.001) without obvious heterogeneity (*I*^2^ = 42.90%, *P*_h_=0.001, Figure 2A). Additionally, similar results were observed with DSS in NSCLC (HR = 2.17, 95% CI = 1.18–3.99, *P*=0.013) without obvious heterogeneity (*I*^2^ = 0.00%, *P*_h_=0.013, Figure 2A). In order to uncover the effect of MASPIN cytoplasm localization on OS, we conducted a subgroup analysis, and the results showed that MASPIN overexpression was associated with poor OS in NSCLC (HR = 1.45, 95% CI = 1.01–2.07, *P*<0.05, Figure 2B) and LUAD (HR = 1.44, 95% CI = 0.33–6.26, *P*=0.626, Figure 2C). In summary, our results showed a statistically negative interaction between MASPIN and NSCLC prognosis, and MASPIN might be a promising prognostic signature in NSCLC.

### Association between MASPIN and clinicopathological characteristics of NSCLC

**Figure 3:**
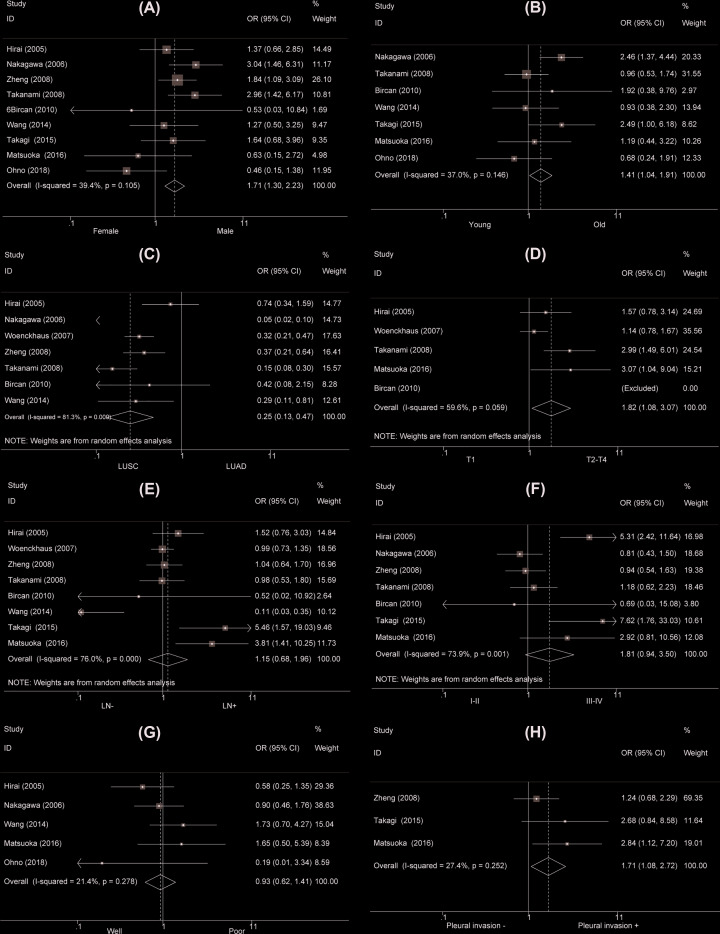
Forest plot for the relationship between total (cytoplasmic + nuclear) MASPIN expression and clinicopathological parameters of NSCLC (**A**) Gender (male versus female); (**B**) age (old versus young); (**C**) type (LUAD versus LUSC); (**D**) T stage (T2–4 versus T1); (**E**) TNM stage (III-IV vs I-II); (**F**) lymph node metastasis (LN+ vs LN−); (**G**) differentiation (poor versus well); (**H**) pleural invasion (yes versus no).

**Figure 4:**
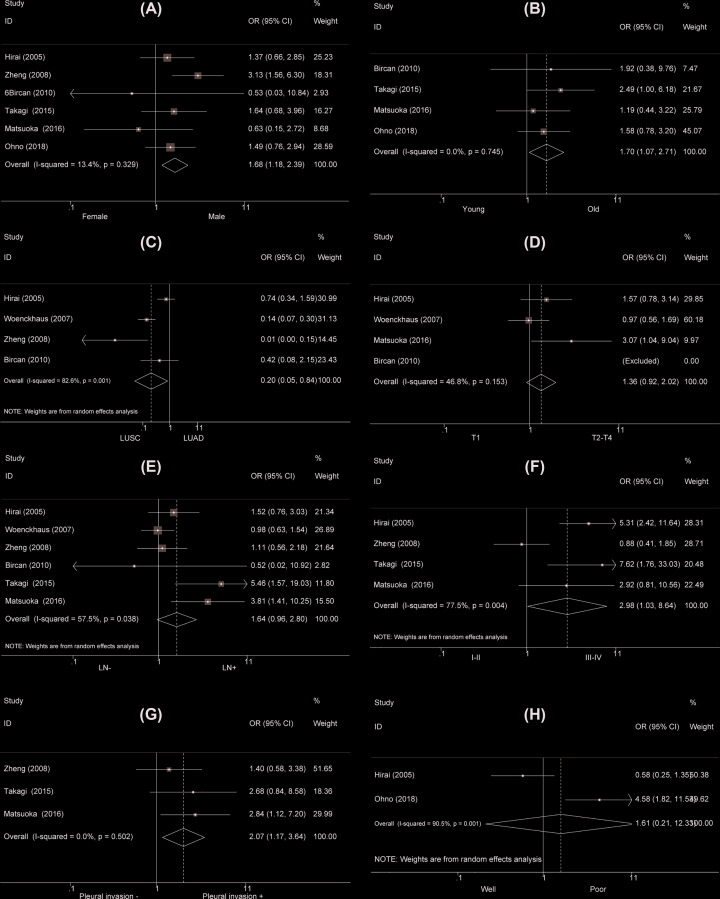
Forest plot for the relationship between cytoplasmic MASPIN expression and clinicopathological parameters of NSCLC (**A**) Gender (male versus female); (**B**) age (old versus young); (**C**) type (LUAD versus LUSC); (**D**) T stage (T2–4 versus T1); (**E**) TNM staging (III-IV vs I-II); (**F**) lymph node metastasis (LN+ vs LN−); (**G**) differentiation (poor versus well); (**H**) pleural invasion (yes versus no).

Not all the included studies discussed the interaction between MASPIN and clinicopathological characteristics of NSCLC. Nine studies showed a higher MASPIN nucleus and cytoplasm expression was detected in male NSCLC patients than female NSCLC patients (OR = 1.71, 95% CI = 1.31–2.23, *P*<0.001, Supplementary Table S2, Figure 3A). The old NSCLC patients had an elevated MASPIN nucleus and cytoplasm expression than young patients (OR = 1.41, 95% CI = 1.04–1.91, *P*=0.027, Supplementary Table S2, Figure 3B). LUSC patients had a lower MASPIN nucleus and cytoplasm expression than LUAD patients (OR = 0.25, 95% CI = 0.13–0.47, *P*<0.001, Supplementary Table S2, Figure 3C). The patients with stage T1 exposed MASPIN nucleus and cytoplasm underexpression compared with those with stages T2–T4 (OR = 1.82, 95% CI = 1.08–3.07, *P*=0.024, Supplementary Table S2, Figure 3D). MASPIN nucleus and cytoplasm expression was not related to clinical stage, differentiation, and lymph node metastasis of NSCLC (OR = 1.81, 95% CI = 0.94–3.50, *P*=0.078; OR = 0.93, 95% CI = 0.62–1.41, *P*=0.74 and OR = 1.15, 95% CI = 0.68–1.96, *P*=0.604, respectively, Supplementary Table S2, Figure 3E–G). Moreover, NSCLC patients with pleural invasion displayed MASPIN nucleus and cytoplasm overexpression than those without with pleural invasion (OR = 1.71, 95% CI = 1.08–2.72, *P*=0.023, Supplementary Table S2, Figure 3H). As for MASPIN cytoplasm expression, the subgroup analysis results were generally consistent with the MASPIN nucleus and cytoplasm expression (Supplementary Table S2, Figure 4).

### Sensitivity analysis and publication bias

A sensitivity analysis of OS was performed to verify the robustness of consequences. The results disclosed there had no significant heterogeneity (Figure 2D). The sensitivity analysis and assessment of publication bias with PFS, DFS, and DSS were not conducted because there were few numbers of included studies. The funnel plot indicated no publication bias analysis of OS (Figure 2E). Moreover, Egger’s test revealed no obvious publication bias (*P*=0.795, Figure 2F).

### The prognostic significances of *SERPINB5* in NSCLC

**Figure 5:**
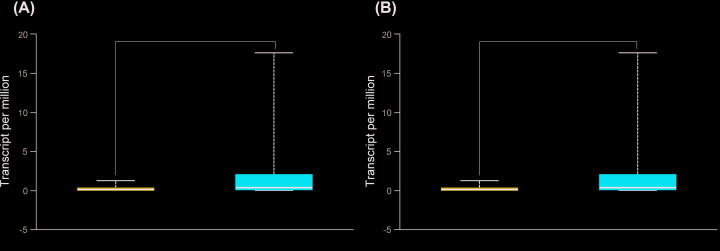
Transcriptional expression of *SERPINB5* in LUAD and LUSC (UALCAN) (**A**) LUAD; (**B**) LUSC. ***P<0.001.

**Figure 6:**
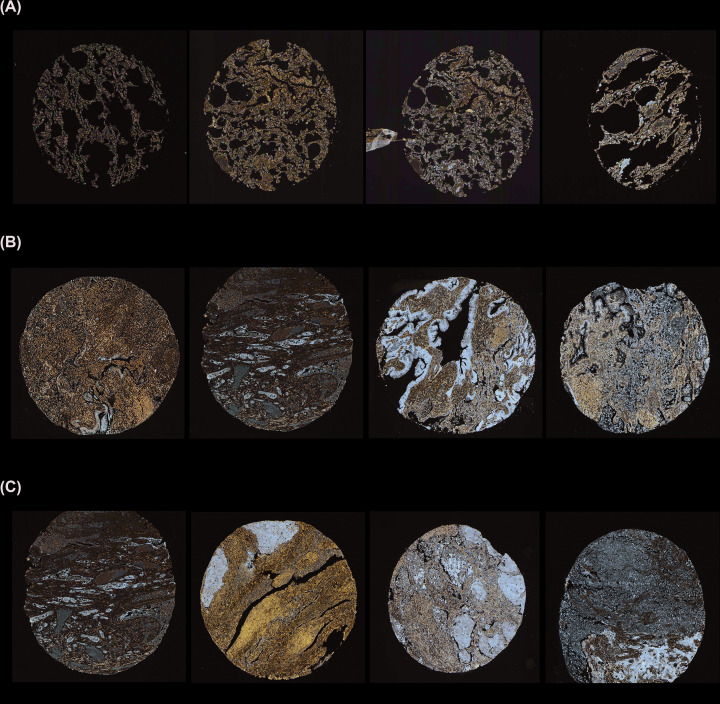
Representative proteins expressions of IHC images of distinct MASPIN were detected in LUAD, LUSC, and normal tissues (Human Protein Atlas) (**A**) MASPIN proteins were found not or low expressed in normal lung tissues. (**B,C**) Significantly high staining expressions were observed in LUAD and LUSC.

**Figure 7:**
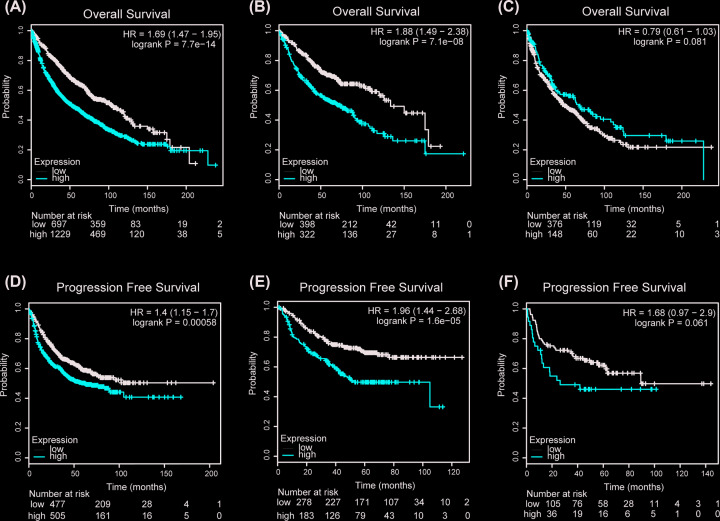
*SERPINB5* expression is correlated with the survival rate of non-small lung cancer patients using the Kaplan–Meier plotter website (**A–C**) The OS rate was related to SERPINB5 mRNA expression in NSCLC, LUAD, and LUSC. (**D–F**) PFS was related to SERPINB5 mRNA expression in NSCLC, LUAD, and LUSC.

The bioinformatics analysis results, perform by UALCAN, showed that *SERPINB5* mRNA expression was higher in both LUAD and LUSC patients than the normal (*P*<0.001, Figure 5). The Human Pathology Atlas illustrated that *SERPINB5* protein expression was significantly higher in both LUAD and LUSC than normal lung (Figure 6). Next, OS was analyzed in NSCLC, LUAD, and LUSC related to *SERPINB5* mRNA expression, and we found that NSCLC and LUAD patients with high expression of *SERPINB5* mRNA had poorer prognosis (HR = 1.69, 95% CI = 1.47–1.95, *P*<0.001; HR = 1.88, 95% CI = 1.49–2.38, *P*<0.001; respectively, Figure 7A,B), and there was no statistically difference in LUSC patients (HR = 0.79, 95% CI = 0.61–1.03, *P*=0.081, Figure 7C). Additionally, the same trend was observed during the PFS analysis (Figure 7D–F).

### The clinicopathological significances of *SERPINB5* in NSCLC

According to Kaplan–Meier plotter, higher *SERPINB5* mRNA expression was negatively correlated with OS of LUSC, T3 and T4 and stage III (*P*<0.05, Table 2), and positively correlated with gender (male and female), smoking and non-smoking, LUAD, T1 and T2, N and stages I and II, (non-) chemotherapy, (non-) radiotherapy and surgical margins negative (*P*<0.05, Table 2). There appeared a positive correlation between *SERPINB5* mRNA overexpression and PFS in gender (male and female), smoking and non-smoking, histology (LUAD and LUSC), T1, N0 and N2, stage I, (non-) chemotherapy, (non-) radiotherapy and surgical margins negative (*P*<0.05, Table 2), whereas negative relationship in T2 and T3, N1 and stage II (*P*<0.05, Table 2).

**Table 2:** The prognostic significance of SERPINB5 mRNA in non-small lung cancer by Kaplan–Meier plotter

Clinicopathological features	OS		PFS	
	HR	*P*	HR	*P*
**Sex**				
Male	1.69 (1.41–2.03)	9.40E-09	1.59 (1.21–2.09)	8.20E-04
Female	1.55 (1.23–1.96)	1.90E-04	1.32 (0.94–1.87)	1.10E-01
**Smoking**				
Yes	1.7 (1.38–2.09)	3.90E-07	1.49 (1.12–1.97)	5.60E-03
No	2.23 (1.27–3.89)	3.90E-03	1.83 (1.13–2.96)	1.30E-02
**Histology**				
Adenocarcinoma	1.88 (1.49–2.38)	7.10E-08	1.96 (1.44–2.68)	1.60E-05
Squamous	0.79 (0.617–1.03)	8.10E-02	1.68 (0.97–2.9)	6.10E-02
**T**				
1	1.71 (1.28–2.3)	2.80E-04	1.49 (0.81–2.75)	2.00E-01
2	1.53 (1.17–1.99)	1.70E-03	0.82 (0.6–1.11)	1.90E-01
3	0.66 (0.37–1.19)	1.67E-01	0.62 (0.22–1.76)	3.60E-01
4	0.64 (0.34–1.23)	1.80E-01		
**N**				
0	1.53 (1.24–1.89)	7.50E-05	1.31 (0.91–1.87)	1.40E-01
1	1.37 (1–1.87)	5.00E-02	0.74 (0.47–1.17)	1.90E-01
2	1.48 (0.91–2.41)	1.10E-01	2.73 (1.22–6.09)	1.10E-02
**TNM stage**				
I	2.08 (1.59–2.72)	4.80E-08	2.05 (1.31–3.2)	1.30E-03
II	1.23 (0.86–1.78)	2.60E-01	0.4 (0.22–0.73)	1.70E-03
III	0.76 (0.43–1.34)	3.40E-01		
**Chemotherapy**				
Yes	2.41 (1.58–3.68)	2.60E-05	1.66 (1.08–2.57)	2.00E-02
No	2.26 (1.62–3.17)	1.00E-06	1.57 (1.03–2.37)	3.30E-02
**Radiotherapy**				
Yes	3.94 (2.2–7.05)	9.30E-07	2.16 (1.24–3.78)	5.70E-03
No	1.76 (1.22–2.53)	2.00E-03	1.37 (0.89–2.12)	1.50E-01
**Surgery success**				
Surgical margins negative	2.25 (1.79–2.84)	1.40E-12	1.85 (1.39–2.46)	2.20E-05

### DNA methylation of *SERPINB5* and its correlation with mRNA expression in NSCLC patients

**Figure 8:**
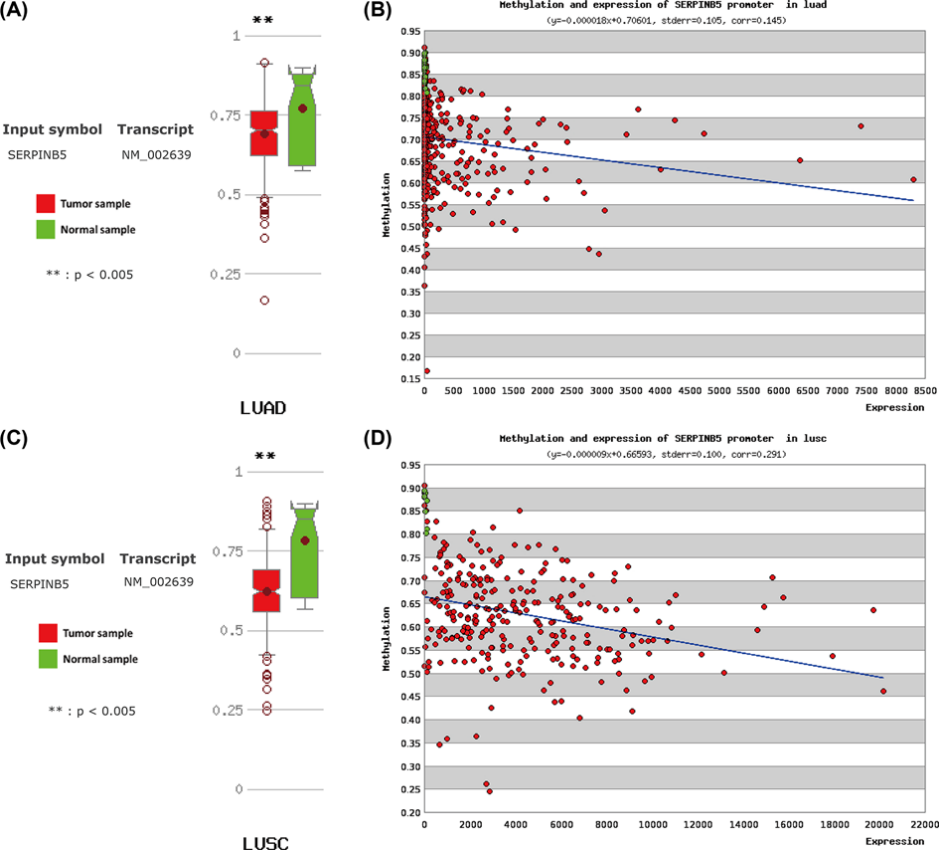
DNA methylation of the *SERPINB5* gene promoter region in non-small lung cancer (MethHC) (**A**) Comparison of DNA methylation levels of the *SERPINB5* gene in LUAD with normal tissues. (**B**) The correlation between DNA methylation and mRNA expression in the *SERPINB5* gene of LUAD. (**C**) Comparison of DNA methylation levels of the *SERPINB5* gene in LUSC with normal tissues. (**D**) The correlation between DNA methylation and mRNA expression in the *SERPINB5* gene of LUSC. Green indicates normal lung tissues, red represents non-small lung cancer tissues.

To recognize the role of methylation in regulating *SERPINB5* expression in NSCLC patients, MethHC was applied to explore the level of DNA methylation in the *SERPINB5* gene promoter region and its correlation with mRNA expression. The difference of the *SERPINB5* methylation level between LUAD and normal samples was statistically significant (*P*<0.005, Figure 8A). Additionally, *SERPINB5* methylation was negatively correlated with its mRNA expression in LUAD (r = −0.128, *P*<0.001, Figure 8B). The statistically significant results were also indicated in LUSC patients (Figure 8C,D).

### Function and pathway enrichment analysis of *SERPINB5*-related genes in LUAD

**Figure 9:**
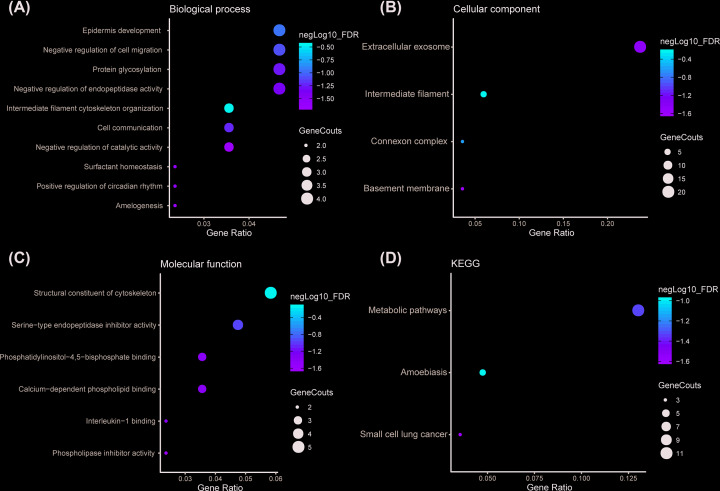
Dot plot of GO enrichment and KEGG pathways analyses from the predicted *SERPINB5*-related genes (**A**) Biological process; (**B**) Cellular component; (**C**) Molecular function; (**D**) KEGG.

To comprehend the functions and potential mechanisms of *SERPINB5* in NSCLC, cBioPortal [25] was used to identify *SERPINB5*-related genes in LUAD. There were 85 genes with Spearman’s correlation coefficients more than 0.4 and were identified as *SERPINB*-related genes in TCGA-LUAD. Besides, 85 genes were put into the DAVID [26,27] for GO and KEGG pathway enrichment analysis, revealing 20 GO terms (Supplementary Table S3) and 3 KEGG pathways (Supplementary Table S4) with statistical significance. These corresponding GO terms were considered as the most specific and useful for describing the concrete function of *SERPINB5* in LUAD, the interaction between different GO terms were visualized as three GO maps (Figure 9A–C). KEGG pathway enrichment analysis results subjected that the genes were enriched in amoebiasis, metabolic pathways, and small cell lung cancer (Figure 9D).

## Discussion

Up to date, more and more studies are emerging to investigate MASPIN expression and its roles in malignant tumors, and carry out in-depth researches on different mechanisms of MASPIN about inhibiting tumor cell plasticity and affecting the context-dependent response to drugs [15]. Nevertheless, only a minority of MASPIN expression and subcellular localization were evaluated in NSCLC and no unanimous agreement was reached. One study announced that MASPIN cytoplasm expression alone could be a useful negative prognostic effect in p-stage IA LUAD [33]. Other studies indicated strong MASPIN nuclear expression was an independent favorable prognostic factor in NSCLC [29,40], and it improved differentiated epithelial phenotypes, decreased tumor angiogenesis, and increased tumor sensitivity to drug-induced apoptosis [41]. In contrast, Bircan et al. indicated MASPIN had no prognostic significance although it was higher in both LUSC and LUAD [42]. There is no doubt that larger studies are urgently needed to reveal the exact role of MASPIN in the occurrence, development, and prognosis of NSCLC. Hence, we summarized 12 studies with 2220 patients, which met preordained inclusion criteria and had high quality according to NOS scores, to obtain more accurate results and further explored the mechanism of MASPIN in NSCLC through different online databases.

Clinical data conflict on the prognostic utility of MASPIN [29,36,43], and we found that MASPIN expression was up-regulated in NSCLC and was closely related to prognosis. Importantly, we not only analyzed the relationship between MASPIN and NSCLC prognosis but also applied different subcellular localization. Our results suggested that MASPIN was statistically correlated with poor prognosis (OS, DFS, and DSS) regarding different subcellular localization (nucleus and cytoplasm or cytoplasm alone), which indicated MASPIN might be a promising prognostic signature in NSCLC, although the nuclear-cytoplasmic expression pattern might have stronger influences on metastasis and proliferation than nuclear expression alone [36]. Our subgroup analysis showed that rising MASPIN protein was associated with poor OS in LUAD patients with no statistical difference (*P*=0.626) while the results from the Kaplan–Meier plotter uncovered that negative correlation between *SERPINB5* mRNA and OS and PFS was statistically significant in LUAD patients (*P*<0.001). In addition, the results of LUAD and LUSC were not identical. The discrepancies might be largely attributable to background heterogeneity, which suggests that larger sample size of clinically relevant studies may be needed to verify this phenomenon. But although LUSC and LUAD account for a large proportion of NSCLC [44], they still do not fully reflect all NSCLC.

In addition, we analyzed the relationship between MASPIN and clinicopathological characteristics from the perspective of meta-analysis and Kaplan–Meier plotter. The results were complementary, as one focused on subcellular localization and the other on prognosis; one is the protein condition and the other is the RNA condition. However, it is found that the expression of both *SERPINB5* protein and mRNA is significantly correlated with various clinicopathological characteristics and survival outcomes. Many studies have revealed the relevant mechanism between MASPIN and the occurrence, development, treatment, and prognosis of lung cancer. In LUAD cells, MASPIN can not only down-regulate the expression of matrix metalloproteinase-2 and integrin β1 to significantly inhibit cell migration and invasion but also affect cell growth mainly by targeting AKT signaling molecules. The different functions of MASPIN may be due to its different molecular characteristics [45]. One study suggested that cytoplasmic MASPIN may play an important role in LUAD by regulating apoptosis and, thus, is a favorable prognostic marker for LUAD patients, whereas the nuclear location may be linked to the promotion of angiogenesis [46]. Others hold MASPIN inhibits cell motility by suppressing Rac1 and PAK1 activity [47], promotes cell adhesion via the PI3K/ERK pathway [47,48], and suppresses survival of lung cancer cells through modulation of AKT pathway [49,50]. It was suggested that the loss-expression of MASPIN may participate in the invasion and metastasis of NSCLC and it has a positive relationship to vasculogenic mimicry in NSCLC [51]. About the treatment, resistance to osimertinib partially arises through MASPIN [52] and a novel statistical methodology for detecting splicing changes in exon array data presented that *SERPINB5* showed alternative splicing in NSCLC patients treated with bevacizumab/erlotinib [53].

To uncover additional mechanisms, we performed methylation analysis. Online databases from MethHC showed that the underlying mechanism of MASPIN affecting NSCLC survival outcomes may be related to hypomethylation and this was following a previous study [54], and this difference may be critical for epigenetic regulation of radiosensitivity [55]. And then, in this study, we demonstrated that *SERPINB5* in both LUAD and LUSC were lowly methylated in the promoter region and that there was an inverse correlation between DNA methylation and mRNA expression. Hypomethylated status means activation of gene expression, therefore, it is not difficult to infer that *SERPINB5* methylation partially contributes to its increased expression and plays an important role in the occurrence and development in NSCLC. Given the statistical difference between *SERPINB5* mRNA and prognosis in LUAD and not in LUAC, TCGA-LUAD was selected for the subsequent analysis of co-expressed genes and enrichment to confirmed that *SERPINB5* influenced related pathways involving the neighboring genes, as is mentioned in a previous study [53,56], and future investigations and validation are needed.

Sensitivity analysis demonstrated that the correlation between MASPIN and NSCLC prognosis was stable and unchanged after removing any one study. Funnel plot and Egger’s test were used to detect publication bias and events indicated no obvious publication bias.

However, the present study was not without limitations. Foremost, all the data analyzed in our study were obtained from other published studies or different online databases, which might cause background heterogeneity, as mentioned earlier. Additionally, eight studies were conducted in Asia (China or Japan) which may result in an excursion in race. Later multinational and multiracial studies for this specific population are needed. Third, the included studies had different definitions of MASPIN cut-off value. Therefore, before carrying out relevant researches, it may be necessary to identify the cut-off value in a unified way. Fourth, according to Kaplan–Meier plotter, higher *SERPINB5* mRNA expression was positively correlated with OS and PFS of surgery success (surgical margins negative) (all *P*<0.0001). Due to the limitations of the data obtained, we could only divide the surgery success into one group, which was bound to cause the loss of information, and the later data with richer dimensions is warranted. Finally, a series of silico-bioinformatic analysis was performed to uncover the promising molecular mechanisms of MASPIN in NSCLC, and more elaborate studies are required to be designed and conducted to validate these results in the future.

In conclusion, our study demonstrated that MASPIN had a statistically negative correlation with NSCLC prognosis, functioning as an oncoprotein by hypomethylation and influencing specific pathways, which lays a foundation for MASPIN to develop into an independent prognostic factor for NSCLC patients.

## Supplementary Material

Supplementary Tables S1-S4

## Abbreviations

CI: confidence interval
DFS: disease-free survival
DSS: disease-specific survival
GO: gene ontology
HR: hazard ratio
KEGG: Kyoto Encyclopedia of Genes and Genomes
LUAD: lung adenocarcinoma
LUSC: lung squamous cell carcinoma
MASPIN: mammary serine protease inhibitor
NOS: Newcastle–Ottawa Scale
NSCLC: non-small cell lung cancer
OS: overall survival
PFS: progression-free survival
*SERPINB5*: human serpin family B member 5
WOS: web of science

## References

[1] Zou Z., Anisowicz A., Hendrix M.J.C. et al. (1994) Maspin, a serpin with tumor-suppressing activity in human mammary epithelial cells. Science 263, 526–529 10.1126/science.8290962 8290962

[2] Berardi R., Morgese F., Onofri A. et al. (2013) Role of maspin in cancer. Clin. Transl. Med. 2, 8 10.1186/2001-1326-2-8 23497644 PMC3602294

[3] Ning J., Yonghong M., Suliang Z. et al. (2002) Maspin sensitizes breast carcinoma cells to induced apoptosis. Oncogene 21, 4089–4098 12037665 10.1038/sj.onc.1205507

[4] Maass N., Ziebart M., Nagasaki K. et al. (2002) Maspin locates to the nucleus in certain cell types. J. Pathol. 197, 274–275 10.1002/path.1103

[5] Bailey C.M., Khalkhali-Ellis Z., Seftor E.A. et al. (2006) Biological functions of maspin. J. Cell. Physiol. 209, 617–624 10.1002/jcp.20782 17001697

[6] Mohsin S.K., Zhang M., Clark G.M. et al. (2003) Maspin expression in invasive breast cancer: association with other prognostic factors. J. Pathol. 199, 432–435 10.1002/path.1319 12635133

[7] Sood A.K., Fletcher M.S., Gruman L.M. et al. (2002) The paradoxical expression of maspin in ovarian carcinoma. Clin. Cancer Res. 8, 2924–2932 12231537

[8] Lonardo F., Li X., Siddiq F. et al. (2006) Maspin nuclear localization is linked to favorable morphological features in pulmonary adenocarcinoma. Lung Cancer 51, 31–39 10.1016/j.lungcan.2005.07.011 16159682

[9] Bray F., Ferlay J., Soerjomataram I. et al. (2018) Global cancer statistics 2018: GLOBOCAN estimates of incidence and mortality worldwide for 36 cancers in 185 countries. CA Cancer J. Clin. 68, 394–424 30207593 10.3322/caac.21492

[10] Antonia S.J., Borghaei H., Ramalingam S.S. et al. (2019) Four-year survival with nivolumab in patients with previously treated advanced non-small-cell lung cancer: a pooled analysis. Lancet Oncol. 20, 1395–1408 10.1016/S1470-2045(19)30407-3 31422028 PMC7193685

[11] Ettinger D.S., Wood D.E., Aggarwal C. et al. (2019) Non-small cell lung cancer, version 4.2019. J. Natl. Compr. Cancer Netw. 12, 1738–1761 10.6004/jnccn.2014.0176 25505215

[12] Hirai K., Koizumi K., Haraguchi S. et al. (2005) Prognostic significance of the tumor suppressor gene maspin in non-small cell lung cancer. Ann. Thorac. Surg. 79, 248–253 10.1016/j.athoracsur.2004.06.118 15620951

[13] Smith S.L., Ratschiller S.G., Watson D., Gugger M. et al. (2003) Maspin - the most commonly-expressed gene of the 18q21.3 serpin cluster in lung cancer - is strongly expressed in preneoplastic bronchial lesions. Oncogene 22, 8677–8687 10.1038/sj.onc.1207127 14647462

[14] Zheng H.-C. and Gong B.-C. (2017) The roles of maspin expression in gastric cancer: a meta- and bioinformatics analysis. Oncotarget 8, 66476–66490 10.18632/oncotarget.20192 29029529 PMC5630429

[15] Sheng S., Bernardo M.M., Dzinic S.H. et al. (2018) Tackling tumor heterogeneity and phenotypic plasticity in cancer precision medicine: our experience and a literature review. Cancer Metastasis Rev. 37, 655–663 10.1007/s10555-018-9767-4 30484007 PMC6853189

[16] Tierney J.F., Stewart L.A., Ghersi D. et al. (2007) Practical methods for incorporating summary time-to-event data into meta-analysis. Trials 8, 16 10.1186/1745-6215-8-16 17555582 PMC1920534

[17] Parmar M.K., Torri V. and Stewart L. (1998) Extracting summary statistics to perform meta-analyses of the published literature for survival endpoints. Stat. Med. 17, 2815–2834 10.1002/(SICI)1097-0258(19981230)17:24<2815::AID-SIM110>3.0.CO;2-8 9921604

[18] Stang A. (2010) Critical evaluation of the Newcastle-Ottawa scale for the assessment of the quality of nonrandomized studies in meta-analyses. Eur. J. Epidemiol. 25, 603–605 10.1007/s10654-010-9491-z 20652370

[19] Chuling F., Hui H. and Zuojun X. (2016) The Newcastle-Ottawa Scale (NOS) for assessing the quality of nonrandomized studies

[20] Liang B., Zhao L.-Z., Liao H.-L. et al. (2019) Rivaroxaban for cancer-associated venous thromboembolism: a systematic review and meta-analysis protocol. Medicine (Baltimore) 98, e18087 10.1097/MD.0000000000018087 31770226 PMC6890290

[21] Chandrashekar D.S., Bashel B., Balasubramanya S.A.H. et al. (2017) UALCAN: a portal for facilitating tumor subgroup gene expression and survival analyses. Neoplasia 19, 649–658 10.1016/j.neo.2017.05.002 28732212 PMC5516091

[22] Asplund A., Edqvist P.-H.D., Schwenk J.M. et al. (2012) Antibodies for profiling the human proteome-The Human Protein Atlas as a resource for cancer research. Proteomics 12, 2067–2077 10.1002/pmic.201100504 22623277

[23] Nagy Á., Lánczky A., Menyhárt O. et al. (2018) Validation of miRNA prognostic power in hepatocellular carcinoma using expression data of independent datasets. Sci. Rep. 8, 9227 10.1038/s41598-018-27521-y 29907753 PMC6003936

[24] Huang W.-Y., Hsu S.-D., Huang H.-Y. et al. (2015) MethHC: a database of DNA methylation and gene expression in human cancer. Nucleic Acids Res. 43, D856–D861 10.1093/nar/gku1151 25398901 PMC4383953

[25] Cerami E., Gao J., Dogrusoz U. et al. (2012) The cBio cancer genomics portal: an open platform for exploring multidimensional cancer genomics data. Cancer Discov. 2, 401–404 10.1158/2159-8290.CD-12-0095 22588877 PMC3956037

[26] Huang D.W., Sherman B.T. and Lempicki R.A. (2009) Systematic and integrative analysis of large gene lists using DAVID bioinformatics resources. Nat. Protoc. 4, 44–57 10.1038/nprot.2008.211 19131956

[27] Huang D.W., Sherman B.T. and Lempicki R.A. (2009) Bioinformatics enrichment tools: paths toward the comprehensive functional analysis of large gene lists. Nucleic Acids Res. 37, 1–13 10.1093/nar/gkn923 19033363 PMC2615629

[28] Yu G.-C., Wang L.-G., Han Y. et al. (2012) clusterProfiler: an R package for comparing biological themes among gene clusters. OMICS 16, 284–287 10.1089/omi.2011.0118 22455463 PMC3339379

[29] Berardi R., Santinelli A., Onofri A. et al. (2012) Maspin expression is a favorable prognostic factor in non-small cell lung cancer. Anal. Quant. Cytol. Histol. 34, 72–78 22611762

[30] Bircan A., Bircan S., Kapucuoglu N. et al. (2010) Maspin, VEGF and p53 expression in small biopsies of primary advanced lung cancer and relationship with clinicopathologic parameters. Pathol. Oncol. Res. 16, 553–561 10.1007/s12253-010-9259-5 20349288

[31] Matsuoka Y., Takagi Y., Nosaka K. et al. (2016) Cytoplasmic expression of maspin predicts unfavourable prognosis in patients with squamous cell carcinoma of the lung. Histopathology 69, 114–120 10.1111/his.12921 27297724

[32] Nakagawa M., Katakura H., Adachi M. et al. (2006) Maspin expression and its clinical significance in non-small cell lung cancer. Ann. Surg. Oncol. 13, 1517–1523 10.1245/s10434-006-9030-z 17009165

[33] Ohno T., Kubouchi Y., Wakahara M. et al. (2018) Clinical significance of subcellular localization of maspin in patients with pathological stage IA lung adenocarcinoma. Anticancer Res. 38, 3001–3007 29715131 10.21873/anticanres.12553

[34] Takagi Y., Matsuoka Y., Shiomi T. et al. (2015) Cytoplasmic maspin expression is a predictor of poor prognosis in patients with lung adenocarcinoma measuring <3 cm. Histopathology 66, 732–739 25322663 10.1111/his.12586

[35] Takanami I., Abiko T. and Koizumi S. (2008) Expression of maspin in non-small-cell lung cancer: correlation with clinical features. Clin. Lung Cancer 9, 361–366 10.3816/CLC.2008.n.052 19073519

[36] Wang X., Wang Y., Li S. et al. (2014) Decreased maspin combined with elevated vascular endothelial growth factor C is associated with poor prognosis in non-small cell lung cancer. Thorac. Cancer 5, 383–390 10.1111/1759-7714.12104 26767029 PMC4704373

[37] Woenckhaus M., Bubendorf L., Dalquen P. et al. (2007) Nuclear and cytoplasmic Maspin expression in primary non-small cell lung cancer. J. Clin. Pathol. 60, 483–486 10.1136/jcp.2005.033407 16698957 PMC1994526

[38] Yaman B., Nart D., Ekren P.K. et al. (2015) Expression of p63, TTF-1 and Maspin in non-small cell lung carcinoma and their effect on the prognosis and differential diagnosis. Turk. Patoloji Derg. 31, 163–174 26456962 10.5146/tjpath.2015.01305

[39] Zheng H.-C., Saito H., Masuda S. et al. (2008) Cytoplasmic and nuclear maspin expression in lung carcinomas: an immunohistochemical study using tissue microarrays. Appl. Immunohistochem. Mol. Morphol. 16, 459–465 18665036 10.1097/PAI.0b013e3181640bb1

[40] Takanami I., Abiko T. and Koizumi S. (2008) Expression of Maspin in non–small-cell lung cancer: correlation with clinical features. Clin. Lung Cancer 9, 361–366 10.3816/CLC.2008.n.052 19073519

[41] Dzinic S.H., Bernardo M.M., Li X. et al. (2017) An essential role of Maspin in embryogenesis and tumor suppression. Cancer Res. 77, 886–896 10.1158/0008-5472.CAN-16-2219 27923833 PMC5313336

[42] Bircan A., Bircan S., Kapucuoglu N. et al. (2010) Maspin, VEGF and p53 expression in small biopsies of primary advanced lung cancer and relationship with clinicopathologic parameters. Pathol. Oncol. Res. 16, 553–561 20349288 10.1007/s12253-010-9259-5

[43] Teoh S.S., Vieusseux J., Prakash M. et al. (2014) Maspin is not required for embryonic development or tumour suppression. Nat. Commun. 5, 3164 10.1038/ncomms4164 24445777 PMC3905777

[44] Skoulidis F. and Heymach J.V. (2019) Co-occurring genomic alterations in non-small-cell lung cancer biology and therapy. Nat. Rev. Cancer 19, 495–509 31406302 10.1038/s41568-019-0179-8 PMC7043073

[45] Zhou J., Hualong Q., Zhou P. et al. (2015) Different maspin functions in the lung adenocarcinoma A549 and SPC-A1 cell lines. Int. J. Mol. Med. 36, 1440–1448 10.3892/ijmm.2015.2336 26329803

[46] Zheng H.-C., Saito H., Masuda S. et al. (2008) Cytoplasmic and nuclear maspin expression in lung carcinomas: an immunohistochemical study using tissue microarrays. Appl. Immunohistochem. Mol. Morphol. 16, 459–465 18665036 10.1097/PAI.0b013e3181640bb1

[47] Odero-Marah V.A., Khalkhali-Ellis Z., Chunthapong J. et al. (2003) Maspin regulates different signaling pathways for motility and adhesion in aggressive breast cancer cells. Cancer Biol. Ther. 2, 398–403 14508113 10.4161/cbt.2.4.471

[48] Jenkinson S.E., Brown L.J., Ombor J. et al. (2017) Identification of novel peptide motifs in the serpin maspin that affect vascular smooth muscle cell function. Biochim. Biophys. Acta Mol. Cell Res. 1864, 336–344 10.1016/j.bbamcr.2016.11.019 27888098

[49] Nam E. and Park C. (2010) Maspin suppresses survival of lung cancer cells through modulation of Akt pathway. Cancer Res. Treat. 42, 42–47 10.4143/crt.2010.42.1.42 20369051 PMC2848751

[50] Zhou J., Hualong Q., Zhou P. et al. (2015) Different maspin functions in the lung adenocarcinoma A549 and SPC-A1 cell lines. Int. J. Mol. Med. 36, 1440–1448 10.3892/ijmm.2015.2336 26329803

[51] Qiao L., Liang N., Zhang J. et al. (2015) Advanced research on vasculogenic mimicry in cancer. J. Cell. Mol. Med. 19, 315–326 10.1111/jcmm.12496 25598425 PMC4407602

[52] Ku B.M., Choi M.K., Sun J.-M. et al. (2018) Acquired resistance to AZD9291 as an upfront treatment is dependent on ERK signaling in a preclinical model. PLoS ONE 13, e0194730 10.1371/journal.pone.0194730 29641535 PMC5895014

[53] Baty F., Klingbiel D., Zappa F. et al. (2015) High-throughput alternative splicing detection using dually constrained correspondence analysis (DCCA). J. Biomed. Inform. 58, 175–185 10.1016/j.jbi.2015.10.002 26483173

[54] Kwon Y.-J., Lee S.J., Koh J.S. et al. (2012) Genome-wide analysis of DNA methylation and the gene expression change in lung cancer. J. Thorac. Oncol. 7, 20–33 10.1097/JTO.0b013e3182307f62 22011669

[55] Kim E.H., Park A.K., Dong S.M. et al. (2010) Global analysis of CpG methylation reveals epigenetic control of the radiosensitivity in lung cancer cell lines. Oncogene 29, 4725–4731 10.1038/onc.2010.223 20531302

[56] Yuan K., Xie K., Fox J. et al. (2013) Decreased levels of miR-224 and the passenger strand of miR-221 increase MBD2, suppressing maspin and promoting colorectal tumor growth and metastasis in mice. Gastroenterology 145, 853–864.e859 10.1053/j.gastro.2013.06.008 23770133 PMC3783518

