# Correction: The roles of MASPIN expression and subcellular localization in non-small cell lung cancer

**DOI:** 10.1042/BSR-20200743_COR

**Published:** 2020-06-30

**Keywords:** MASPIN, SERPINB5, non-small cell lung cancer, prognosis

The authors of the original article above article “The roles of MASPIN expression and subcellular localization in non-small cell lung cancer” (Bioscience Reports (2020) 40(5), DOI: 10.1042/BSR20200743) would like to correct Figure 6B, as the IHC images of LUAD and LUSC were repeated due to mis-operation in visualisation. The authors had mistakenly reused the image of LUSC (Antibody CAB009570) for LUAD (Antibody HPA019025). The authors declare that these corrections do not change the results or conclusions of this paper, and express their sincere apologies for any inconvenience that this error has caused to the readers. The corrected version of Figure 6 is presented here.

**Figure 6:**
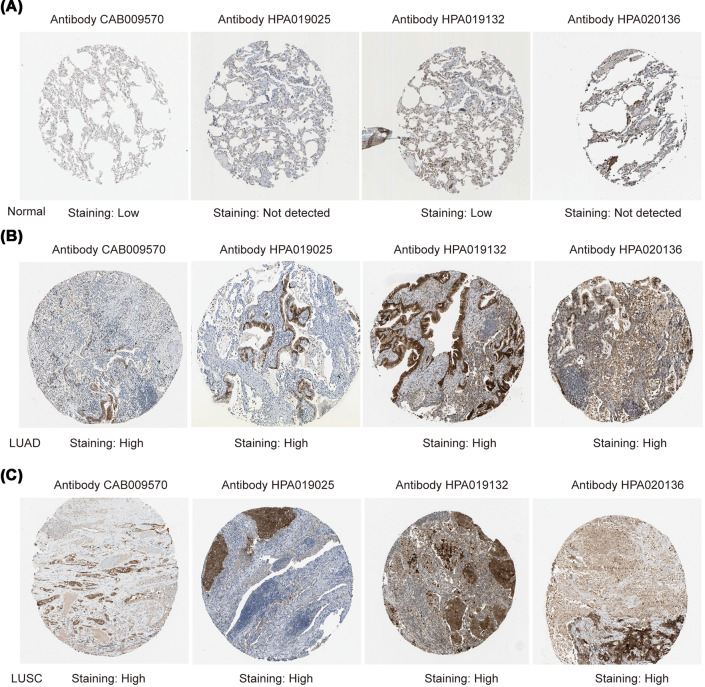
Representative proteins expressions of IHC images of distinct MASPIN were detected in LUAD, LUSC, and normal tissues (Human Protein Atlas) (**A**) MASPIN proteins were found not or low expressed in normal lung tissues. (**B,C**) Significantly high staining expressions were observed in LUAD and LUSC.

