# Supplementary material for: The roles of MASPIN expression and subcellular localization in non-small cell lung cancer
Source: Biosci Rep. 2020 May 26;40(5):BSR20200743. doi: 10.1042/BSR20200743 (PMC7251327; doi:10.1042/BSR20200743)
Supplement: Supplementary Tables S1-S4 [file BSR-2020-0743_supp.pdf]

Supplementary Table S1. Search strategies in Medline.

#1	"Carcinoma, Non-Small-Cell Lung" [Mesh]
#2	Carcinoma, Non Small Cell Lung [All fields] OR Lung Carcinomas, Non-Small-Cell [All fields] OR Non Small Cell Lung Carcinoma [All fields] OR Non-Small Cell Lung Cancer [All fields]
#3	#1 OR #2
#4	"SERPIN-B5" [Supplementary Concept]
#5	Maspin [All fields] OR SERPINB5 [All fields] OR Protease inhibitor 5 [All fields]
#6	#4 OR #5
#7	"Prognosis" [Mesh]
#8	Prognoses [All fields] OR Prognostic Factors [All fields] OR Factor, Prognostic [All fields]
#9	#7 OR #8
#10	#3 AND #6 AND #9

Supplementary Table S2. Meta-analysis of the association between cytoplasmic or total (cytoplasmic + nuclear) MASPIN expression and clinicopathological characteristics.

**Location**	**Clinicopathological parameter**	**Studies (n)**	**OR (95% CI)**	***P*-value**	**I^2(%)**	**Model**
**T(C+N)**	Gender (male versus female)	9	1.71(1.31-2.23)	**<0.001**	39.40%	Fixed
**T(C+N)**	Age (old versus young)	7	1.41(1.04-1.91)	**0.027**	37.00%	Fixed
**T(C+N)**	Type (LUAD versus LUSC)	7	0.25(0.13-0.47)	**<0.001**	81.30%	Random
**T(C+N)**	T stage (T2-4 versus T1)	5	1.82(1.08-3.07)	**0.024**	59.60%	Random
**T(C+N)**	Clinical stage (III-IV versus I-II)	7	1.81(0.94-3.50)	0.078	73.90%	Random
**T(C+N)**	LN (yes versus no)	8	1.15(0.68-1.96)	0.604	76.00%	Random
**T(C+N)**	Differentiation (poor versus well)	5	0.93(0.62-1.41)	0.74	21.40%	Fixed
**T(C+N)**	Pleural invasion (yes versus no)	3	1.71(1.08-2.72)	**0.023**	27.40%	Fixed
**C**	Gender (male versus female)	6	1.68(1.18-2.39)	**0.004**	13.40%	Fixed
**C**	Age (old versus young)	4	1.7(1.07-2.71)	**0.026**	0.00%	Fixed
**C**	Type (LUAD versus LUSC)	4	0.20(0.05-0.84)	**0.028**	82.60%	Random
**C**	T stage (T2-4 versus T1)	4	1.36(0.92-2.02)	0.127	46.80%	Fixed
**C**	TNM (III/IV versus I/II)	4	2.98(1.03-8.64)	**0.045**	88.40%	Random
**C**	LN (yes versus no)	6	1.64(0.96-2.81)	0.068	57.50%	Random
**C**	Differentiation (poor versus well)	2	1.61(0.21-12.33)	0.65	90.50%	Random
**C**	Pleural invasion (yes versus no)	3	2.07(1.18-3.64)	**0.012**	0.00%	Fixed

Abbreviation: LUAD: lung adenocarcinoma; LUSC: lung squamous cell carcinoma; NSCLC: non-small lung cancer; LN: lymph node; OS: overall survival; PFS: progression-free survival; DFS: disease-free survival; DSS: disease-specific survival; OR: odds ratio; HR: hazard ratio; C: cytoplasm; N: nucleus; T: total, T = C+N; CI: confidence interval.

Supplementary Table S3. GO functional annotation of the most significant related targets of MASPIN.

GO ID	GO term	Count (%)	Gene symbol	*P*
**Biological process**				
GO:0045104	Intermediate filament cytoskeleton organization	3	KRT6C, KRT6A, KRT16	2.04E-03
GO:0008544	Epidermis development	4	KRT17, KRT16, GJB5, LAMC2	6.75E-03
GO:0030336	Negative regulation of cell migration	4	KRT16, PKP2, NKX2-1, SRGAP1	9.15E-03
GO:0007154	Cell communication	3	GJB3, GJB4, GJB5	1.28E-02
GO:0006486	Protein glycosylation	4	ST3GAL5, B3GNT5, B3GALT5, GALNT11	1.46E-02
GO:0010951	Negative regulation of endopeptidase activity	4	PI3, SERPINB7, SERPINB4, SERPINB3	1.75E-02
GO:0042753	Positive regulation of circadian rhythm	2	NKX2-1, ARNTL2	3.56E-02
GO:0043129	Surfactant homeostasis	2	ADGRF5, NAPSA	3.56E-02
GO:0097186	Amelogenesis	2	ITGA6, PERP	4.00E-02
GO:0043086	Negative regulation of catalytic activity	3	SERPINB3, ANXA2, ANXA2P2	4.54E-02
**Cellular component**				
GO:0005882	Intermediate filament	5	KRT6C, KRT6A, KRT17, KRT16, PKP2	1.47E-03
GO:000592	Connexon complex	3	GJB3, GJB4, GJB5	3.69E-03
GO:0070062	Extracellular exosome	20	KRT6C, GDA, KRT6A, RAB3B, KRT6B, STXBP1, C16ORF89, BCAM, NAPSA, SDCBP2, ANXA2, ANXA2P2, GPD1L, KRT17, KRT16, AKR1B10, SLC2A1, PI3, SERPINB4, SERPINB3	2.94E-02
GO:0005604	Basement membrane	3	ITGA6, ANXA2, ANXA2P2	4.61E-02
**Molecular function**				
GO:0005200	Structural constituent of cytoskeleton	5	KRT6A, KRT6B, KRT17, KRT16, HIP1	1.12E-03
GO:0004867	Serine-type endopeptidase inhibitor activity	4	PI3, SERPINB7, SERPINB4, SERPINB3	7.62E-03
GO:0005546	Phosphatidylinositol-4,5-bisphosphate binding	3	ANXA8, ANXA2, HIP1	2.41E-02

GO:0005544	Calcium-dependent phospholipid binding	3	ANXA8, ANXA2, ANXA2P2	2.41E-02
GO:0019966	Interleukin-1 binding	2	IL1R2, TRIM16	2.46E-02
GO:0004859	Phospholipase inhibitor activity	2	ANXA2, ANXA2P2	4.47E-02

Supplementary Table S4. KEGG pathway enrichment analysis of MASPIN target genes

KEGG ID	KEGG term	Count (%)	Gene symbol	*P*
hsa05146	Amoebiasis	4	IL1R2, SERPINB4, LAMC2, SERPINB3	9.71E-03
hsa01100	Metabolic pathways	11	GDA, ST3GAL5, B3GNT5, B3GALT5, AKR1B10, PLCD3, CYP4F3, UCK2, COX6B2, ACAD8, GALNT11	2.28E-02
hsa05222	Small cell lung cancer	3	ITGA6, CDK6, LAMC2	4.94E-02
